# Associations Between Posterior Communicating Artery Aneurysms and Morphological Characteristics of Surrounding Arteries

**DOI:** 10.3389/fneur.2022.874466

**Published:** 2022-07-13

**Authors:** Weili Hao, Hong Hao, Chun-Feng Ren, Xiangling Wang, Bulang Gao

**Affiliations:** ^1^Department of Medical Research, Shijiazhuang People's Hospital, Shijiazhuang, China; ^2^The First Affiliated Hospital of Zhengzhou University, Zhengzhou, China; ^3^Department of Catheterization Room, Shijiazhuang People's Hospital, Shijiazhuang, China

**Keywords:** posterior communicating artery aneurysm, internal carotid artery, morphological parameters, tortuosity, angle

## Abstract

**Objectives:**

To explore the associations between posterior communicating artery (PComA) aneurysms and morphological characteristics of arteries upstream of and around the PComA bifurcation site.

**Methods:**

In this study, fifty-seven patients with PComA aneurysms and sixty-two control subjects without aneurysms were enrolled. The centerlines of the internal carotid artery (ICA) and important branches were generated for the measurement and analysis of morphological parameters, such as carotid siphon types, diameters of two fitting circles, and the angle formed by them (D_1_, D_2_, and ϕ), length (L) and tortuosity (T_L_) of ICA segment between an ophthalmic artery and PComA bifurcations, bifurcation angle (θ), tortuosity (T_ICA_ and T_PComA_), and flow direction changes (θ_ICA_ and θ_PComA_) around the PComA bifurcation site.

**Results:**

No significant difference (*p* > 0.05) was found in the siphon types (*p* = 0.467) or L (*p* = 0.114). Significant differences (*p* < 0.05) were detected in D_1_ (*p* = 0.036), T_L_ (*p* < 0.001), D_2_ (*p* = 0.004), ϕ (*p* = 0.008), θ (*p* = 0.001), T_ICA_ (*p* < 0.001), T_PComA_ (*p* = 0.012), θ_ICA_ (*p* < 0.001), and θ_PComA_ (*p* < 0.001) between the two groups. T_ICA_ had the largest area under the curve (*AUC*) (0.843) in the receiver operating characteristic (*ROC*) analysis in diagnosing the probability of PComA aneurysms presence and was identified as the only potent morphological parameter (*OR* = 11.909) associated with PComA aneurysms presence.

**Conclusions:**

The high tortuosity of the ICA segment around the PComA bifurcation is associated with PComA aneurysm presence.

## Introduction

Intracranial aneurysms are frequently found in the region of the Willis circle and its associated branches (1), especially at the anterior communicating cerebral artery junction, the internal carotid-posterior communicating artery junction, and the middle cerebral artery bifurcation (1). The most common outcome of aneurysm rupture is subarachnoid hemorrhage, which may result in high mortality and permanent morbidity (2). Hypertension, smoking, genetics, and female sex have been identified as risk factors for the development of intracranial aneurysms (2), and hemodynamic factors which are primarily dependent on vascular morphology have also been reported to play a key role in aneurysm development (3). The geometry of the parent artery affects the hemodynamics in both the parent artery and the aneurysm (4–8), and in particular, the arterial morphology at the aneurysmal site and the upstream vasculature are possibly associated with the pathogenesis of aneurysm formation. The posterior communicating artery (PComA), originating from the posterolateral surface of the internal carotid artery (ICA) after the carotid siphon and ophthalmic segment, connects the posterior with the anterior cerebral circulations (9). PComA aneurysms account for 15–25% of intracranial aneurysms (10), and their rupture risk is significantly higher than those of middle cerebral artery bifurcation aneurysms (11). It was hypothesized that some morphological characteristics that might contribute to the initiation and development of the PComA aneurysm existed around the PComA origin, and this study was consequently performed to investigate the associations between the morphological characteristics of the ICA and PComA and the presence of PComA aneurysms.

## Materials and Methods

This cross-sectional case-control study was approved by the Scientific Research Ethics Committee of Shijiazhuang People's Hospital, and all patients had provided written informed consent to participate. Image data of consecutive patients who underwent digital subtraction angiography (DSA) between September 2016 and April 2020 in our hospital were collected. All methods were performed in accordance with the relevant guidelines and regulations. The inclusion criteria were patients older than 40 years but younger than 80 years, with high-quality images of the ICA, ophthalmic artery (OphA), and PComA. PComA aneurysms were referred to the aneurysm on the bifurcation site of the ICA-PComA junction anatomically. Patients with poor-quality images affecting correct analysis, multiple or large aneurysms, and fetal-type posterior cerebral artery were all excluded.

**Figure 1:**
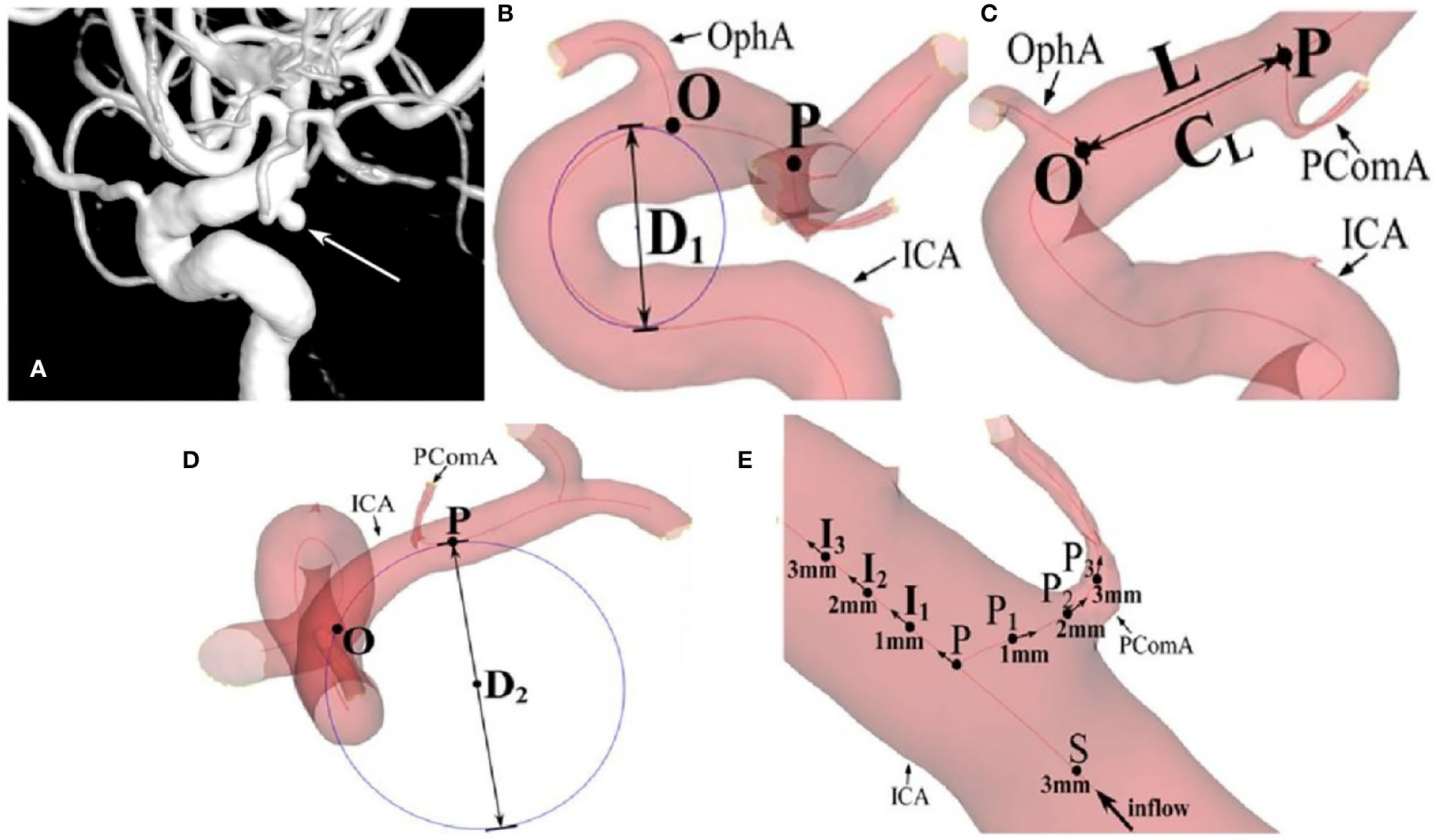
The measurement of morphological parameters. **(A)** A posterior communicating artery (PComA) aneurysm is shown (arrow). **(B)** D_1_ is the diameter of the incircle fitting the carotid siphon bend. **(C)** L is the length of the ICA segment from OphA bifurcation (Point O) to PComA bifurcation (Point P), and T_L_ referred to its tortuosity. **(D)** D_2_ refers to the diameter of the osculating circle fitting L. **(E)** θ is defined as the bifurcation angle between the internal carotid artery (ICA) and PComA, formed by Points I_1_, P, and P_1_. T_ICA_ is defined as the average tortuosity of segments SI_2_ and SI_3_ along ICA, and the T_PComA_ is the average tortuosity of three segments SP_2_ and SP_3_ along the blood flow. θ_ICA_ refers to the average flow direction changes of PI_2_ and PI_3_, and θ_PComA_ refers to the average flow direction changes of PP_2_ and PP_3_.

Using Siemens workstation software (Siemens, Munich, Germany), raw three-dimensional rotational angiography data were reconstructed and exported in the stereolithography (STL) format for the measurement of the morphological parameters in the Mimics software (Materialize, Belgium). After the vessels were set as partial transparent, the centerlines of ICA and important branches were generated automatically, bifurcation points were clearly displayed, and morphological parameters were measured based on the centerlines (Figure 1 and Table 1). According to the anatomical shape, the carotid siphon was further classified into types U, V, C, or S similar to the approach used by Zhang et al. (12) and measured with modification. The length of the ICA segment from the OphA bifurcation (Point O) to the PComA bifurcation (Point P) was named L, and T_L_ referred to its tortuosity. Tortuosity of certain segment is calculated by the formula of 1 – (linear distance/distance along the branch). D_1_ was the diameter of the incircle fitting the carotid siphon bend, D_2_ referred to the diameter of the osculating circle fitting L, and ϕ was the intersection angle formed by the aforementioned two fitting circle planes. Point S, about 3 mm distal from Point P on the upstream ICA, was considered as the starting point of tortuosity measurement of certain segments around Point P. Along the ICA blood flow, Point I_1_, I_2_, and I_3_ were 1, 2, and 3 mm distal from Point P, respectively. In the same way, Point P_1_, P_2_, and P_3_ were 1, 2, and 3 mm away from Point P on the PComA centerline. Thus, Points I_1_, P, and P_1_ formed angle θ, which was defined as the bifurcation angle between ICA and PComA. T_ICA_ was defined as the average value of the tortuosity of segments SI_2_ and SI_3_ along ICA, and T_PComA_ was the average value of the tortuosity of segments SP_2_ and SP_3_ along the PComA. The tangent direction of each indicated point was regarded as the blood flow direction at this site, and thus, the angle between the tangent directions of Point P and I_2_ represented the direction change of blood flow from Point P to I_2_. The direction changes of blood flow from Point P to other points of I_3_, P_2_, and P_3_ were also calculated in a similar way. Hence, θ_ICA_ referred to the average flow direction changes of PI_2_ and PI_3_, and θ_PComA_ referred to the average flow direction changes of PP_2_ and PP_3_.

**Table 1:** Definition of the morphological parameters.

**Parameters**	**Definition**
D_1_ (mm)	Diameter of the incircle fitting the carotid siphon bend
L (mm)	Length of the ICA segment from ophthalmic artery bifurcation to posterior communicating artery bifurcation
T_L_	Tortuosity of L
D_2_ (mm)	Diameter of the osculating circle fitting L
ϕ (°)	Intersection angle of the aforementioned two fitting circle planes
θ(°)	Bifurcation angle, formed by Points I_1_, P and P_1_
T_ICA_	Average tortuosity of segments SI_2_ and SI_3_ along ICA
T_PComA_	Average tortuosity of segments SP_2_ and SP_3_ along the blood flow
θ_ICA_ (°)	Average changes of flow direction from P to I_2_ and I_3_
θ_PComA_ (°)	Average changes of flow direction from P to P_2_ and P_3_

### Statistical Analysis

All statistical analyses were performed using the SPSS software (version22, IBM, Chicago, IL, USA). Normal distribution measurement data were recorded as “$\overset{\bar{\;}}{x}$ ± *s*” (mean ± standard deviation [SD]) and tested with the independent *t*-test for comparison of the mean values, and non-normal distribution measurement data were presented as *M (P*_25_*, P*_75_*)* (median (interquartile range [IQR])) and tested with the *Mann-Whitney U-*test for the comparison of the median values. Enumeration data were presented as numbers and percentages and tested with the χ^2^-test. The receiver operating characteristic (*ROC*) curve and binary logistic regression analysis were carried out to find the underlying relationships between the morphological characteristics and PComA aneurysms presence. The value of *p* < 0.05 was considered as statistically significant.

## Results

A total of 119 patients were enrolled, including 57 patients with PComA aneurysms (16 men and 41 women with a mean age of 62.84 ± 8.54 years) and 62 control subjects without cerebral aneurysms (28 men and 34 women with a mean age of 59.82 ± 9.17 years) (Table 2). There were no difference in age (*t* = −1.816 and *p* = 0.072), gender ratio (χ^2^ = 3.723 and *p* = 0.054), smoking (χ^2^ = 2.053 and *p* = 0.152), drinking (χ^2^ = 0.052 and *p* = 0.470), heart disease (χ^2^ = 0.018 and *p* = 0.894), and hyperlipemia (χ^2^ = 0.047 and *p* = 0.829), but a significant difference was found in the hypertension (χ^2^ = 5.977 and *p* = 0.014) and diabetes (χ^2^ = 7.184 and *p* = 0.007) between the two groups.

**Table 2:** Univariate analysis of data between the control and posterior communicating artery (PComA) aneurysm groups.

**Variables**	**Control (*n* = 62)**	**PComA aneurysm (*n* = 57)**	**statistics**	***P***
Male (*n*, %)	28 (45.2%)	16 (28.1%)	3.723	0.054^a^
Age (years)	59.82 ± 9.17	62.77 ± 8.49	−1.816	0.072^b^
Hypertension	31 (50.0%)	41 (71.9%)	5.977	0.014^a^
Smoking	11(17.7%)	5(8.8%)	2.053	0.152^a^
Drinking	8(12.9%)	5(8.8%)	0.521	0.470^a^
Diabetes	12(19.4%)	2(3.5%)	7.184	0.007^a^
Heart disease	7(11.3%)	6(10.5%)	0.018	0.894^a^
Hyperlipemia	5(8.1%)	4(7%)	0.047	0.829^a^
Rupture	-	42(73.7%)	-	-
Siphon type (*n*, %)			2.543	0.467^a^
C	18 (29.0%)	20 (35.5%)		
S	14 (22.6%)	15 (26.3%)		
U	17 (27.4%)	16 (28.1%)		
V	13 (21.0%)	6 (10.5%)		
D_1_ (mm)	7.99 ± 1.56	8.81 ± 2.00	−2.118	0.036^b^
L (mm)	9.42 ± 2.32	10.12 ± 2.35	−1.593	0.114^b^
T_L_	0.08 ± 0.04	0.12 ± 0.05	−4.444	<0.001^b^
D_2_ (mm)	13.30 (11.13, 17.81)	11.75 (10.12, 13.78)	−2.886	0.004^c^
ϕ (°)	67.63 ± 25.86	54.51 ± 21.51	2.697	0.008^b^
θ (°)	97.42 ± 13.09	105.59 (98.62, 118.13)	3.271	0.001^c^
T_ICA_	0.03 (0.02, 0.04)	0.06 (0.04, 0.13)	5.546	<0.001^c^
T_PComA_	0.30 ± 0.09	0.37 (0.28, 0.41)	2.524	0.012^c^
θ_ICA_ (°)	21.04 (16.52, 25.38)	45.86 ± 23.76	5.963	<0.001^c^
θ_PComA_ (°)	96.95 ± 17.93	114.76 ± 21.76	−4.989	<0.001^b^

*Data were presented as “mean ± standard deviation (SD)”, median (interquartile range, IQR) or numbers and percentages of patients. (a) χ^2^-test; (b), t-test; and (c), Mann–Whitney U-test*.

**Figure 2:**
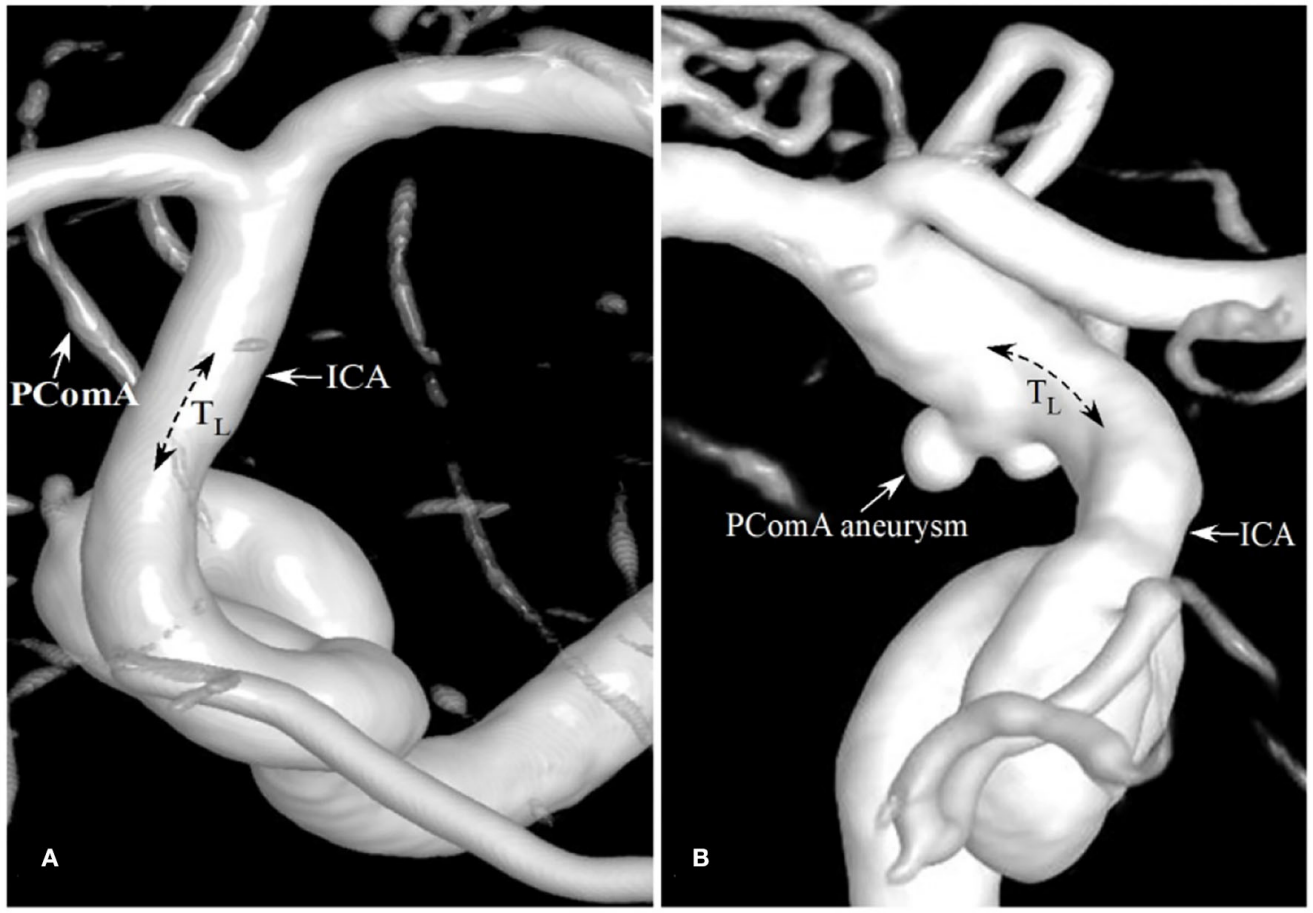
Patients with the different degrees of tortuosity (T_ICA_) of the PComA segment of the ICA. **(A)** A patient with no PComA aneurysms had the T_ICA_ tortuosity of 0 at the PComA segment of ICA. **(B)** A patient with a PComA aneurysm had the T_ICA_ of 0.05.

A univariate analysis was performed to check the significant differences in the parameters between the two groups (Table 2). There was no statistical difference in the siphon types between the two groups (χ^2^ = 2.543 and *p* = 0.467), yet D_1_ of the PComA aneurysm group was significantly larger than that of the control group (8.81 ± 2.00 mm vs. 7.99 ± 1.56 mm, *t* = −2.118, *p* = 0.036). No statistical difference was observed in L (the distance from the OphA bifurcation to the PComA bifurcation) between the two groups (9.42 ± 2.32 mm for the control group and 10.12 ± 2.35 mm for the aneurysmal group, *t* = −1.593, and *p* = 0.114). However, T_L_ was significantly higher in the PComA aneurysm group than in the control group (0.12 ± 0.05 vs. 0.08 ± 0.04, *t* = −4.444, and *p* < 0.001). The ϕ of PComA aneurysm group was significantly smaller than that of the control group (54.51 ± 21.51 degrees vs. 67.63 ± 25.86 degrees, *t* = 2.697, and *p* = 0.008), so was D_2_ between the two groups (13.30 mm vs. 11.75 mm, *t*=-2.886, and *P*=0.004). The θ of the PComA aneurysm group was significantly larger than that of the control group (105.59 vs. 97.42 degrees, *z* = 3.271, and *p* = 0.001). T_ICA_ and T_PComA_ of the aneurysmal group were significantly larger than those of the control group (0.06 vs. 0.03, *z* = 5.546, *p* < 0.001 for T_ICA_; 0.37 vs. 0.30, *z* = 2.524, *p* = 0.012 for T_PComA_, respectively, Figure 2). Both θ_ICA_ and θ_PComA_, describing changes of the blood flow direction from Point P to certain points of each branch, were significantly larger in the aneurysmal group than those in the control group (45.86 ± 23.76 vs. 21.04, *z* = 5.963, *p* < 0.001 for θ_ICA_, and 114.76 ± 21.76 vs. 96.95 ± 17.93, *t* = −4.989, *p* < 0.001 for θ_PComA_).

An *ROC* curve analysis was carried out to analyze the potentiality of the morphological parameters in diagnosing the probability of the presence of PComA aneurysms (Table 3), such as D_1_, T_L_, D_2_, ϕ, θ, T_ICA_, T_PComA_, θ_ICA_, and θ_PComA_, which were found to have significant (*p* < 0.05) differences between the PComA aneurysm and control group in the univariate analysis. The T_ICA_ had the largest *AUC* (0.843, 95% *CI* 0.762–0.963) than any other parameters, whereas the *AUCs* of the two parameters of the ICA (0.843 for T_ICA_ and 0.827 for θ_ICA_) were both larger than those of the PComA (0.650 for T_PComA_ and 0.747 for θ_PComA_). The cutoff point of T_ICA_ was 0.525, and we thus changed T_ICA_ from a continuous variable to a categorical variable in the subsequent logistic regression analysis (T_ICA_ values < 0.525 were recorded as low and > 0.525 as high).

**Table 3:** The receiver operating characteristic (ROC) curve analysis of morphological parameters for the diagnosing presence of PComA aneurysms.

**Variables**	**AUC**	**Standard error**	***P***	**95% CI**	
				**Lower limit**	**Upper limit**
D_1_	0.622	0.058	0.041	0.509	0.735
T_L_	0.725	0.051	<0.001	0.625	0.826
D_2_	0.357	0.057	0.016	0.246	0.468
ϕ	0.360	0.056	0.019	0.249	0.470
θ	0.705	0.054	0.001	0.599	0.812
T_ICA_	0.843	0.041	<0.001	0.762	0.923
T_PComA_	0.650	0.059	0.012	0.536	0.765
θ_ICA_	0.827	0.044	<0.001	0.740	0.914
θ_PComA_	0.747	0.051	<0.001	0.647	0.848

*AUC, area under the curve; CI, confidence interval*.

There were strong significant correlations between T_ICA_ and θ_ICA_ and between T_PComA_ and θ_PComA_ (Table 4). The θ_ICA_ and θ_PComA_ were thus excluded in the logistic regression analysis to eliminate collinearity. The T_ICA_ and T_PComA_, together with other parameters of hypertension, D_1_, T_L_, D_2_, ϕ, and θ which were significantly different (*p* < 0.05) between the PComA aneurysm and the control group in the univariate analysis, were included in the binary logistic regression analysis (Table 5). Only T_ICA_ was identified as the most potent factor associated with the presence of PComA aneurysms (*b* = 2.477, *p* < 0.001). As *b* > 0, T_ICA_ was a risk factor for PComA aneurysms presence. In other words, the probability of PComA aneurysms presence for patients with T_ICA_ >0.525 were 11.909 times those with T_ICA_ >0.525 (odds ratio [*OR*] = 11.909, 95% *CI* 3.224–43.993).

**Table 4:** Pearson's correlation between tortuosity and angles.

**Variables**	**θ_ICA_**	**θ_PComA_**
T_ICA_	*r* = 0.846, *P* < 0.001	–
T_PComA_	–	*R* = 0.819, *P* < 0.001

**Table 5:** Binary logistic regression analysis of morphological parameters associated with PComA aneurysm presence.

**Variables**	**b**	**Standard error**	***P***	**OR**	**95% CI**	
					**Lower limit**	**Upper limit**
Hypertension	0.702	0.638	0.272	2.017	0.577	7.050
Diabetes	−1.212	1.636	0.201	0.236	0.046	1.906
D_1_	0.210	0.188	0.265	1.233	0.853	1.782
T_L_	10.813	7.855	0.169	49656.149	0.010	2.411E+11
D_2_	−0.041	0.061	0.509	0.960	0.851	1.083
ϕ	−0.003	0.013	0.816	0.997	0.972	1.023
θ	0.003	0.025	0.913	1.003	0.955	1.053
T_ICA_	2.477	0.667	<0.001	11.909	3.224	43.993
T_PComA_	2.168	3.922	0.580	8.738	0.004	19053.468
constant	−4.347	3.128	0.165	0.013		

*OR, odds ratio; CI, confidence interval*.

## Discussion

Cerebral aneurysms, usually occurring at arterial junctions, bifurcations, or bends with abrupt angles where excessive hemodynamic stresses are exerted on the arterial walls (5, 6, 8, 13–18), have been increasingly detected in the general population because of the fast development of medical imaging techniques. Knowledge of the association between vasculature characteristics and the presence of cerebral aneurysms can assist in understanding the pathogenesis of aneurysms (5, 6, 8, 13–18). This is why the relationship was investigated between PComA aneurysm presence and relevant cerebral vascular morphology. It was found that arterial tortuosity, especially tortuosity of the ICA segment around PComA bifurcation, contributed to the presence of PComA aneurysms.

Because the inflow from the parent artery upstream can affect the intra-aneurysmal hemodynamics, the upstream portion of the parent vessel of cerebral aneurysms should be included to accurately investigate the intra-aneurysmal hemodynamics and affecting factors (4). Moreover, the carotid siphon types (S, U, etc.) may assist in evaluating the risk of aneurysm pathology in a specific patient (19), and a narrower carotid siphon was assumed to promote aneurysm formation in susceptible individuals (20). The carotid siphon at the upstream of the parent artery was included in our study to investigate its role in affecting the downstream PComA aneurysm presence. However, no significant difference was found in the carotid siphon types in relation to the PComA aneurysm presence. Thus, the upstream parent artery segment closer to the PComA origin was given more attention and included in the study. As the relatively short supreclinoid segment was associated with the development of PComA aneurysm (21), the ICA segment from the OphA origin to the PComA origin (L) together with its tortuosity (T_L_) were compared between the aneurysm and control subjects. Although the length L was not significantly different between the two groups, T_L_ was significantly greater in the patients with aneurysmal than in the control subjects, which suggests that the tortuosity of the adjacent upstream artery may be associated with the presence of PComA aneurysms.

After studying the simulated fluid dynamics in two successive bends, Kee et al. found that the latter bend influences the outflow pattern to varying degrees depending on the orientation of its upstream bend (22). The study by Zhang et al. (12) demonstrated that the larger angle between the carotid siphon bend and its former ICA bend could lead to disadvantageous hemodynamic factors for lesion formation. In light of these results, the intersection angle, ϕ, formed by two circles (one fitted the carotid siphon bend, and the other fitted the ICA segment L) representing two bends upstream to the PComA origin, was measured in our study to investigate the role of upstream blood flow direction change in affecting aneurysm presence. Data analysis showed that the ϕ in the aneurysm group was significantly smaller than that in the control group, inferring that the smaller ϕ may be involved in the development of an aneurysm. A smaller ϕ suggests abrupt changes in the flow direction, while a larger ϕ indicates slow and little changes in the blood flow direction.

It has been proven that the angle formed between the ICA and PComA was significantly larger in patients with aneurysms than controls (23), so was the PComA bifurcation angle θ as demonstrated in our study. Unfortunately, θ did not show statistical significance in the logistic regression analysis. On the other hand, measurement of the intersection angle is often of great subjectivity due to the visual angles among different operators in practice, whereas tortuosity measurement is simpler comparatively. To eliminate the collinearity effect, tortuosity values other than bifurcation angles were used in the logistic regression analysis. Finally, only T_ICA_ was identified as a statistically significant risk factor of PComA aneurysm presence. Therefore, it is T_ICA_ rather than the bifurcation angle θ that contributes to PComA aneurysms presence. Measurement of tortuosity took vascular segments around the PComA bifurcation site into consideration, which could better describe the key morphological characteristics around the PComA bifurcation site. In this study, the T_ICA_ of the aneurysmal group was significantly larger than that of the control group, and this was in accordance with previous studies proving that highly-curved arteries have been shown to cause greater hemodynamic stresses and subsequent initiation, growth, and rupture of aneurysms (7, 24–26). It has been revealed that changes in blood flow direction caused by curvatures of the ICA carotid siphon can alter the originally linear blood flow into turbulent flow (27), and this could explain the fact that ICA bends harboring ruptured cerebral aneurysms possess a higher curvature than ICA bends without ruptured aneurysms (28). In the studies by Gao et al. (14–16), stent deployment at arterial bifurcations to treat bifurcation aneurysms had been demonstrated to cause immediate and delayed bifurcation angular narrowing (14), decreased region of direct flow impingement, and reduced total pressure and wall shear stress at the bifurcation apex wall (15, 16). These studies suggest that changes in the vascular morphology will alter the blood flow hemodynamics to initiate aneurysms. Thus, changes in the potent morphological parameters, T_ICA_, may be accompanied by alterations in hemodynamic stresses at the bifurcation apex wall to initiate an aneurysm. This may be the potential mechanism for inducing aneurysm presence. In summary, these studies suggest that arterial curvature is associated with aneurysm initiation and evolution, which supports the outcomes of our study.

In our study, the *AUC* of T_ICA_ and θ_ICA_ were larger than those of T_PComA_ and θ_PComA_ in the *ROC* analysis in diagnosing the probability of the presence of PComA aneurysms. These findings indicate that with regard to asymmetrical arterial branches, morphologic characters (T_ICA_ and θ_ICA_) of the ICA segment around the PComA bifurcation contribute more to PComA aneurysm presence than those of the PComA parameters (T_PComA_ and θ_PComA_). Hence to a certain degree, this can explain the fact that almost all ICA aneurysms were considered sidewall aneurysms in some studies (7), as they originate either directly from the ICA or from the origin of a branch that is much smaller than the ICA, such as the ophthalmic, anterior choroidal, and posterior communicating arteries.

Posterior communicating artery has an unusual characteristic in that there is an abrupt curve immediately after the originating site. The high incidence of PComA aneurysms may be associated with the particular anatomic structure and abnormal hemodynamic conditions at the ICA-PComA segment (1). So, identifying risk factors for these aneurysms could be conducive to clinical practice in predicting the possible presence of PComA aneurysm in some suspect populations, thus beneficial to the prevention and treatment of PComA aneurysms. In the present study, T_ICA_ of the aneurysmal group was significantly larger than those of the control group, with the largest *AUC* in *ROC* curve analysis, and was finally identified as a risk factor of PComA aneurysm presence in the logistic regression analysis. Although hypertension as a risk factor for the development of intracranial aneurysms (2) was significantly different (*p* < 0.05) between the PComA aneurysm and control groups in the univariate analysis, it did not enter the final logistic regression model, which shed light on morphology characteristics in PComA aneurysm pathomechanism superior to the demographics. Diabetes mellitus, which was also significantly different between the two groups did not enter the final logistic regression analysis. This may indicate that hypertension and diabetes mellitus may affect the patients in other aspects than the vascular morphological parameters of the PComA, even though further studies are necessary to clarify this point. This is because no current studies have revealed that these two factors can affect the morphological parameters of the PComA. Therefore, T_ICA_ served as a stable independent indictor to be significantly associated with PComA aneurysm presence. Furthermore, tortuosity measurement is more convenient than the measurement of intersection angle in practice, thus favoring imaging diagnosis.

In the clinical practice of treating cerebral aneurysms, a stent can be deployed to assist coil embolization of cerebral aneurysms. The deployment of an intracranial stent can significantly decrease the arterial angle and hemodynamic stresses, which is beneficial to thrombosis within the aneurysm cavity and complete occlusion of the aneurysm (14–16). Since our current study revealed that the high tortuosity of the ICA segment around the PComA bifurcation is associated with PComA aneurysm presence, an intracranial stent can also be deployed at this segment to treat aneurysms, and deployment of the stent will certainly straighten the ICA segment and decrease the tortuosity, which is beneficial to complete occlusion and healing of the aneurysm. Moreover, the high tortuosity of the ICA segment around the PComA bifurcation may remind the physician to perform angiographic follow-up to closely monitor possible aneurysm formation at this segment so as to prevent the life-threatening event of aneurysm rupture and subarachnoid hemorrhage.

This study had some limitations, such as the retrospective and one-center study design, data of Chinese patients enrolled only, non-randomization, a small cohort of patients, and lack of computational fluid dynamics analysis, which may all affect the outcomes and the generalization of this study. Moreover, “intra-patient” comparison was not possible to compare the aneurysmal side and normal side in one same patient for a similar analysis of the morphological factors in affecting aneurysm formation because not all patients had the three-dimensional angiographic data for such analysis. Future studies will have to resolve these issues for better outcomes.

In conclusion, the high tortuosity of the ICA segment around PComA bifurcation, could contribute to PComA aneurysm presence.

## Data Availability Statement

The raw data supporting the conclusions of this article will be made available by the authors, without undue reservation.

## Ethics Statement

The studies involving human participants were reviewed and approved by the Ethics Committee of Shijiazhuang People's Hospital. The patients/participants provided their written informed consent to participate in this study.

## Author Contributions

WH and BG: Study design and data analysis. XW, WH, HH, XZ, and C-FR: Data collection. XZ: Study supervision. WH and XZ: Writing of the original version. BG: Revision of the original. All authors approved the article and agreed to be accountable for all aspects of this work.

## Funding

This study was supported by grants from the Hebei Health Department Plan (No. 20190163) and the China National Natural Scientific Funding (No. 81901184).

## Conflict of Interest

The authors declare that the research was conducted in the absence of any commercial or financial relationships that could be construed as a potential conflict of interest.

## Publisher's Note

All claims expressed in this article are solely those of the authors and do not necessarily represent those of their affiliated organizations, or those of the publisher, the editors and the reviewers. Any product that may be evaluated in this article, or claim that may be made by its manufacturer, is not guaranteed or endorsed by the publisher.
